# Dermatology workforce over a decade in Saudi Arabia: demographics, distributions, and future challenges

**DOI:** 10.1186/s12960-022-00725-0

**Published:** 2022-03-28

**Authors:** Abdulrahman Alfawzan, Saad Altalhab, Mohammad Alkhowailed

**Affiliations:** 1 Division of Dermatology, Ministry of National Guard Health Affairs, Riyadh, Saudi Arabia; 2 King Abdullah International Medical Research Center, Riyadh, Saudi Arabia; 3 College of Medicine, King Saud Bin Abdulaziz University for Health Sciences, Riyadh, Saudi Arabia; 4 Department of Dermatology, College of Medicine, Imam Mohammad Ibn Saud Islamic University, Riyadh, Saudi Arabia; 5 Department of Dermatology, College of Medicine, Qassim University, Buraidah Qassim, Saudi Arabia

**Keywords:** Dermatology workforce, Dermatologists, Saudi Arabia

## Abstract

**Background:**

The dermatology workforce is an important topic, as many countries are facing an undersupply of dermatologists, while some are expecting a surplus. Therefore, we conducted this study to identify the current dermatology workforce demographics in Saudi Arabia (SA) and the changes in such demographics over the last 10 years to identify future workforce-related challenges.

**Methods:**

This study was conducted in SA, and it included all the practicing dermatologists in the country over the last decade (2010–2020). The number of practicing dermatologists, their gender, their nationality, and dermatology residency candidates and graduates were obtained from the Saudi Commission for Health Specialties (SCFHS). The geographic distribution of dermatologists was obtained from the Ministry of Health Statistical Yearbook 2018.

**Results:**

As of September 2020, there were 2678 practicing dermatologists in SA at a ratio of 7.82 dermatologists per 100 000 people. Of the 2678 dermatologists, only 24.8% were Saudis. The Saudi dermatologist ratio has been almost constant over 10 years, ranging from 1.3 to 1.9 per 100 000 people. Of all Saudi dermatologists, 42% were female. The number of residents who graduated from the residency program was not consistent for each year and ranged from 4 to 25. The number of dermatologists varied by region, with 9.2 in Riyadh and 3.4 in Najran per 100 000 people.

**Conclusions:**

The results of our study revealed that a quarter of dermatologists in SA are Saudis. In addition, the number of non-Saudi dermatologists has increased in the last 10 years, while the number of Saudi dermatologists to the population has remained almost constant. There is also a geographic maldistribution of dermatologists, with urban areas having a higher number of dermatologists than rural areas. We encourage local studies that can elucidate the factors influencing the workforce, such as the dermatologist appointment waiting time, dermatologists’ working hours, and the geographic maldistribution of dermatologists in the country.

## Introduction

The current dermatology workforce status remains an important topic across the world, as many countries are facing an undersupply of dermatologists, and some are expecting a surplus. In addition, the demand for dermatologists is increasing worldwide for many reasons, including the increasing incidence of skin cancers and many other dermatological inflammatory diseases [1]. Furthermore, the expansion of dermatology practices into the surgical, procedural, and medical fields has required more dermatologists in the workforce to match the demand. Another related problem that needs to be addressed is the geographic maldistribution of dermatologists in many countries. It has been shown that dermatologists prefer to work in urban areas, which has resulted in a shortage of dermatologists in rural areas [2, 3].

Saudi Arabia (SA) has one of the fastest growing populations worldwide. As such, more adequately trained and specialized healthcare providers are in high demand to meet people’s healthcare needs, especially because the population is also an aging one. The most recent study about the dermatology workforce in SA was conducted by Bin Saif et al. in 2010 [4]. They reported that the number of dermatologists per 100 000 people was 3.76, in 2007 with only 1.16 being Saudi. However, it has been more than a decade since then, and the healthcare system in SA has undergone a huge transformational change over that time. This has raised many questions regarding the sufficiency of trained dermatologists to meet the population’s demands, the geographic distribution of dermatologists around the country, and the expectations of achieving adequate specialized care in the future. Therefore, we conducted this study to identify the current dermatology workforce demographics of SA, the changes that have transpired over the last 10 years, and the future challenges to such a workforce.

## Materials and methods

This study was conducted among dermatologists practicing in SA from January 2010 to September 2020. SA is located in the Arabian Peninsula and is made up of 13 regions: Riyadh, Makkah, Madinah, Qassim, Eastern Region, Asir, Tabuk, Hail, the Northern Border, Jazan, Najran, Al-Bahah, and Al-Jawf. The population of SA as of 2019, according to the General Authority for Statistics, is 34 218 169 [5].

The Saudi Commission for Health Specialties (SCFHS), which is a scientific commission, is responsible for developing, supervising, and evaluating health-related training programs and related examinations as well as issuing professional certifications. It is also responsible for setting controls and standards for the practices of health professions and for recommending general plans to develop human resources for health [6]. SCFHS classifies postgraduate training programs into three groups of the highest quality training to lowest quality training. Of the certificates classified in the first group are the Saudi specialty certificate, specialization certificates from the Royal College of Physicians and Surgeons of Canada, and American Board Certification, while the second group includes the Kuwait and Jordan Boards and many others (Table 1) [7]. To obtain the data needed for this study, an official e-mail was sent to SCFHS, on September 12, 2020, in request of the following data: (1) the number of dermatologists and all physicians currently practicing in SA, (2) the number of dermatologists practicing in SA by year from 2010 to 2020, (3) the number of Saudi and non-Saudi dermatologists currently practicing in SA, (4) the number of male and female dermatologists among the dermatologists currently practicing in SA, (5) the current number of dermatologists at each level from residents to consultants, and (6) the number of dermatology residency program graduates in each year from 2010 to 2020. In addition, data about the geographic distribution of dermatologists currently practicing in the country were obtained from the Saudi Ministry of Health’s (MOH) *Health Statistical Yearbook 2018* [8]. As the data that were requested did not include the names and other personal information of any identified person or were merely accessed through the internet, institutional review board (IRB) approval was not required. The data on the current population of SA were obtained from the *Statistical Yearbook 2018* of the General Authority for Statistics [9].

**Table 1:** Ranks of physicians in Saudi Arabia according to the SCFHS

Rank	Description
Consultant	A physician who has a certificate from the first or the second group and has acquired the necessary experience and the other classification requirements
Senior registrar	A health care provider who has acquired the postgraduate training program certificate from the first or second group, such as a Saudi board certificate
Registrar	A health care provider who has acquired a certificate from the third group and completed the required qualifications
Training resident	A medical student who is enrolled in a postgraduate training program belonging to the first (Saudi board certificate or equivalent) and second groups
Resident	A medical student who is enrolled in postgraduate training and has a certificate from the third group and did not complete the requirements to qualify as a registrar or has experience in the specialty field of not less than 2 years

*SCFHS* Saudi Commission for Health Specialties

Data entry and processing were conducted using Microsoft Excel 2016. The ratio of dermatologists to the population was calculated by dividing the number of dermatologists by the entire population and then multiplying the result by 100 000.

## Results

At the time of calculations, the number of registered dermatologists in SA was 2678, with an estimated 7.82 dermatologists per 100 000 people. Almost half of the dermatologists practicing in the country were registrars (46.8%, n = 1254), and 96% of them were non-Saudis. Of all the dermatologists in the country, only 24.8% (n = 664) were Saudis, but of the 18.8% (n = 504) of all dermatologists who were consultants, 71.8% (n = 362) were Saudis. There were 105 training residents, and only 3 were non-Saudis. The number of female residents was double that of male residents. For every five consultants, there was one training resident. Most Saudi dermatologists were male, while most non-Saudi dermatologists were female. More information is shown in Table 2.

**Table 2:** Number of registered dermatologists in Saudi Arabia by rank (as of September 2020)

Rank	Saudi		Non-Saudi		Total (%)	
	Male	Female	Male	Female (%)		
Consultant	249	113	89	53	504 (18.82)	
Senior registrar	62	52	82	147	343 (12.81)	
Registrar	22	29	460	743	1254 (46.82)	
Training resident	34	68	1	2	105 (3.92)	
Resident	17	18	49	388	472 (17.62)	
Total	384	280	681	1333	2678	100%

**Fig. 1:**
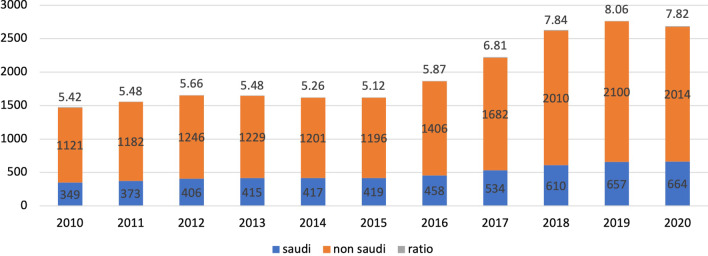
Saudi to non-Saudi dermatologists by year and ratio to 100 000 population

Figure 1 shows the number of Saudi and non-Saudi dermatologists in SA over the last 10 years. The number of dermatologists per 100 000 people in 2020 was 7.82. It grew from 5.42 in 2010 to 8.06 in 2019. The rate of increase in the number of Saudi dermatologists has not been steady and slow compared to that of non-Saudi dermatologists. The most pronounced growth in Saudi dermatologists was seen in 2017 and 2018, with 76 new dermatologists each year. However, the ratio of Saudi dermatologists to the population increased from 1.3 to 1.9 in the past 10 years, while the ratio of non-Saudi dermatologists increased from 4.1 to 5.9 in the same period, with an increase in the actual number from 1121 to 2014 over the 10-year period. The most pronounced increase in the number of non-Saudi dermatologists in the country was from 2017 to 2019.

**Fig. 2:**
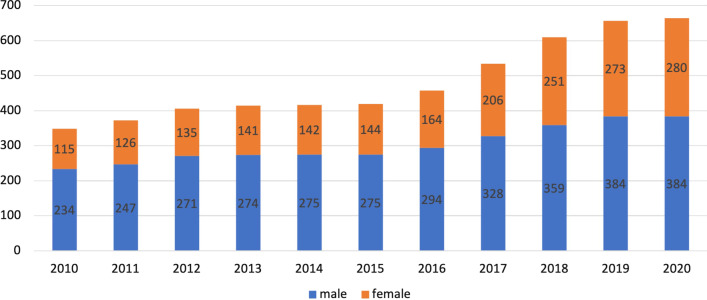
Number of Saudi male and female dermatologists by year

Figure 2 shows the number of male and female Saudi dermatologists by year within the past 10 years. As of September 2020, there were 664 registered Saudi dermatologists practicing in SA. In 2010, the ratio of male-to-female dermatologists was 2:1. Over 10 years, the percentage of female Saudi dermatologists increased from 32 to 42%, particularly from 2015 to 2020. As of September 2020, the number of female dermatologists in the country was 280, while the number of male dermatologists was 384.

Table 3 shows the number of doctors in SA who graduated from and were matched to a dermatology program from 2010 to 2020. Dermatology programs consist of 4 years of training (from R1 to R4). The number of dermatology graduates has been up and down over the 10-year period, while the number of matched residents has almost doubled over the same period. In 2010, 20 residents were matched to a residency program. After 4 years, only 15 graduated from the program. The most recent batch who graduated from the program in 2019 was 8, while the number of matched residents 4 years ago in 2015 was 34.

**Table 3:** Numbers of doctors who were matched to and graduated from a dermatology residency program from 2010 to 2019

Years	Matched	Graduated
2010	20	4
2011	18	25
2012	21	10
2013	22	7
2014	27	15
2015	34	5
2016	32	10
2017	36	7
2018	36	11
2019	34	8

**Fig. 3:**
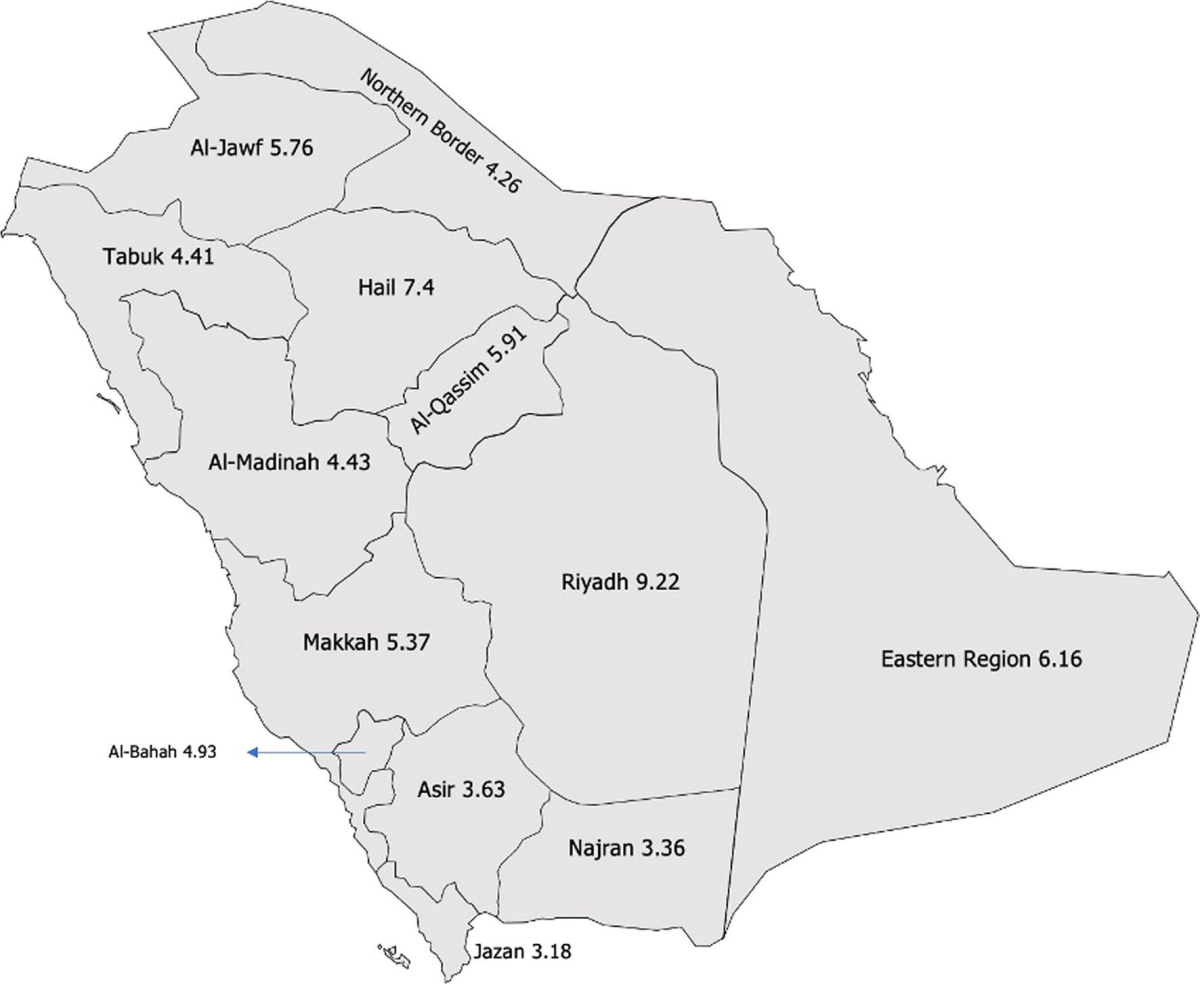
Dermatologists per 100 000 people in Saudi Arabia, 2018

In 2018, the dermatologists practicing in Saudi MOH hospitals and the private-sector dermatologists represented 89.8% of all dermatologists in SA. The remaining 10.2% were registered in other governmental sectors, such as the National Guard Health Affairs. Figure 3 shows the geographic distribution of dermatologists practicing in Saudi MOH hospitals and private-sector dermatologists per 100 000 people in SA in 2018. The ratio of dermatologists ranged from 3.3 to 9.2 across the 13 regions. The most populated regions in SA are Makkah and Riyadh, and the highest number of dermatologists in 2018 was in Riyadh (9.2). Of this number, 8.1 worked in the private sector. The lowest ratios were 3.18, 3.36, and 3.63 (in Jazan, Najran, and Asir, respectively) per 100 000 people. The regions with a population lower than 1 000 000 (Tabuk, Hail, Najran, Al-Jawf, Al-Bahah, and Northern Borders) had higher rates of dermatologists practicing in MOH hospitals.

## Discussion

### Increasing number of dermatologists over the last decade

In this study, we identified the current dermatology workforce distribution, demographics, and changes in SA over the last 10 years. We found that there has been a growth in the total number of dermatologists in SA over the last decade. This increasing number of dermatologists could be attributed to the increased demand for dermatologists over this period. One of the reasons for the increasing demand is the increased prevalence of dermatology-related diseases, such as skin cancers, worldwide and locally [10]. Locally, despite all the protective factors, such as traditional Saudi protective clothing (Thob, Abayah, and Scarf), the lower attitude in the Arabian Peninsula, and the lower rate of ultraviolet (UV) light exposure, the incidence of skin cancers has been increasing [11, 12]. This increased incidence has been attributed to the higher number of qualified dermatologists treating patients and the improved Saudi Cancer Registry reports. According to the latest Saudi Cancer Registry report (2017), there were 555 new cases of non-melanoma skin cancer in SA in 2017; there were only 427 in 2014. As for new cases of melanoma skin cancer in SA, they rose from 41 in 2014 to 50 in 2017. Although SA lacks a national registry for dermatological diseases, several single-center studies have suggested that SA has a high prevalence of dermatological diseases, such as skin appendages disorders, dermatitis, pigmentary disorders, and skin infections [13]. In fact, the most recent single-center studies on dermatitis showed a higher prevalence than older studies [13]. Also, dermatologists provide care for people with diseases associated with dermatological manifestations [14, 15].

Another factor for the increase in the number of dermatologists in SA over the past 10 years is the expansion of the popularity of cosmetic dermatology in the country within this period. Many young adults have considered undergoing minor cosmetic procedures [16, 17]. In a study involving primary care patients, almost half of the study participants were willing to undergo a minor cosmetic procedure [16]. Social media, whose use has increased in SA in the past decade, is also an important factor influencing the dermatology workforce in the country. In a study conducted among 816 female Saudi college students, 48.5% admitted to having considered undergoing a cosmetic procedure due to social media influence [17], and of the 142 students in this study who underwent a cosmetic procedure, 119 (84%) were influenced by social media [17].

### Number of dermatologists worldwide

The World Health Organization (WHO) recommends that human resources for health needs be estimated based on workloads rather than by physician/population ratio [18]. The Royal College of Physicians estimated the number of needed consultants in England based on data about workload, referrals, and working hours of consultants from 2009 to 2010 government statistics. They concluded that they needed 1.6 dermatology consultants per 100 000 people [19]. However, many countries have estimated supply and demand according to the physician/population ratio due to a lack of information about physicians’ work hours, workloads, credentials, and wages. Brazil is planning to meet this demand by having 1.2/100 000 dermatologists/population [20]. The number of researchers and teaching assistants who usually work fewer hours needs to be considered when estimating the number of dermatologists in the population. In the United States, the proposed number of dermatologists needed to meet the population demand is 4 per 100 000 [21]. The most recently reported number of dermatologists in the United States and Brazil was 3.4 and 3.5 per 100 000 people, respectively [20, 21]. In the United States, although the number of practicing dermatologists increased over the 10-year period from 2005 to 2014, the dermatologist appointment waiting time did not change [22]. In addition, around a third of dermatologists think that there is a shortage of dermatologists in their respective areas [22]. This has been reinforced by the fact that 70% of section code areas have fewer than 4 dermatologists per 100 000 people [21]. As such, they depend on advanced physician assistants (APAs) and family physicians to provide dermatological care to patients. From 2005 to 2014, the employment of APAs, such as physician assistants and nurse practitioners in the United States, increased from 28 to 46% [22]. This is not true for SA, where there has been a lower number of family physicians and APAs, which may explain the relatively higher number of dermatologists therein (7.82/100 000) [23].

### Saudi dermatologists

Of all the practicing dermatologists in SA, 24.8% are Saudis. Thus, there are 1.9 dermatologists for every 100 000 people, an increase from 2007, when the ratio was 1.2 dermatologists to 100 000 people [4]. Surprisingly, the ratio of Saudi dermatologists to the population has remained almost constant over the last 20 years. The lack of local dermatologists has been maintained over the years, perhaps because SA has one of the fastest-growing populations in the world and has a limited number of training positions in the field of dermatology [24]. The Saudi Dermatology and Venereology Program, under the supervision of the SCFHS, is one of the best dermatology training programs in the region. It consists of 4 years of training, with the first year in different medical specialties. The program provides the highest level of training, teaching sessions, and research under the supervision of expert consultants and academic professors. The training program is carried out through patient-side training, education, and interactive teaching sessions; meetings to discuss the latest literature in the field; and other extracurricular activities, such as conferences and workshops [25]. There were four graduates from the first batch of trainees in 1999. In the past decade, the number of graduates has remained constant, although the number of matched residents has doubled. This indicates that residents spend more years in the training program than they should.

### Female dermatologists

In many countries, most dermatologists are female. In the United States, the number of Board-certified female dermatologists of total dermatologists increased from 24% in 1992 to 52.5% in 2017 [26]. In Brazil, 78.4% of dermatologists are female [20]. This gender shift also applies to training residents. Almost two-thirds (64.1%) of dermatology training residents in the United States are female [27]. This has not been the case in SA, where only 29% of all dermatologists in 2007 were female. Over the last decade, however, the number of female dermatologists has grown from 32 to 42% of total practicing dermatologists in SA. This is attributed to the higher number of women enrolled in higher education institutions seeking job opportunities. In addition, this shift is expected to continue to increase in the future; thus, the number of female dermatologists is expected to surpass the number of male dermatologists. One of the recommendations of the WHO is to ensure balanced gender opportunities for eligible high school graduates to advance into health training programs [18]. More female dermatologists are needed in the workforce, as it has been shown that female patients are more likely to prefer having a female doctor [28]. This is reinforced by the cultural and religious backgrounds of SA. In addition, a meta-analysis showed that female doctors accommodate their patients for a longer time compared to male doctors, and female doctors are more likely to involve their patients in decision-making and show patients more empathy [29]. In the United States, female doctors work fewer hours and see fewer patients compared to their male counterparts [30]. There have been no local studies on the working hours or number of patients per doctor in SA in general or by gender.

### Foreign physicians and the geographic distribution of dermatologists

Of all dermatologists practicing in SA, 75% are non-Saudis. Since the 1980s, SA has experienced a dramatic expansion in all living standards, including healthcare. To compensate for the deficit in the number of Saudi healthcare providers, many foreign healthcare workers have come to the country. Their efforts and contributions have been and continue to be crucial to the continuity of the highest quality healthcare in SA. Furthermore, the number of foreign physicians has increased to 64.4% of all practicing physicians in the country. However, dependency on foreign physicians has many disadvantages. They have a high rate of sudden turnover and departure, which would increase an institution’s costs in terms of hiring and training other physicians for certain positions [31]. In addition, language is an important barrier. In a study of 16 foreign physicians, most foreign physicians without an Arabic background had language difficulties in the initial 2–3 years in the country [32]. Cultural differences between countries are also very important, since in SA, the culture differs considerably from that of other parts of the world [32]. In the past 10 years, the rate of increase of Saudi physicians to non-Saudi physicians was 19.5%, while it was only 1% for Saudi dermatologists.

One of the problems related to the dermatology workforce most often encountered worldwide is geographic maldistribution. That is, dermatologists generally prefer to practice in urban areas rather than in rural areas. This can be attributed to the higher number of job opportunities available to dermatologists in urban areas [2, 21]. In addition, many prefer to work in an academic hospital or in the same area, where they are trained for various reasons, one of which is proximity to their families’ residences. The emergence of cosmetic dermatology also drives more dermatologists to urban areas, as there is a higher demand for cosmetic procedures [33, 34]. In the United States, 38.6% of the entire dermatology workforce is situated in only 100 of the 712 area code sections nationwide [21]. This has also been observed in SA: the number of dermatologists per population practicing in Riyadh is three times that in other areas, namely, Najran, Asir, and Jazan. These three areas are the only areas out of 13 that have fewer than the recommended number of dermatologists to meet the demands of the population (4 dermatologists per 100 000 people) [21]. This has resulted in less access to dermatological healthcare in those areas, which in turn has resulted in poorer treatment outcomes and higher costs, because patients have to travel to urban areas to get the care that they need. A possible solution to the geographic maldistribution of dermatologists in SA is teledermatology. Perhaps the best benefit of teledermatology is its expansion of dermatologic care to rural areas [35, 36]. In the current COVID-19 pandemic, many hospitals, including the major medical cities in SA, are utilizing teledermatology to ensure the safety of both patients and doctors and to provide the best care to patients. We recommend that through this experience, we learn the pros and cons of teledermatology and develop it to generalize its usage, especially for improving healthcare access in rural areas. Perhaps higher compensation for dermatologists working in rural areas will result in a higher employment rate of medical practitioners in rural areas as well. Increasing living standards and investing in infrastructure will strongly encourage health workers to work in rural areas [37].

The WHO produced a number of documents addressing physician maldistribution and the employment of foreign physicians [18, 37]. According to the WHO Global Strategy on Human Resources for Health: Workforce 2030, they are planning to decrease dependency on foreign physicians by half for all countries by 2030. This can be accomplished by training more physicians, employing them, and ensuring that a country retains them. Finally, they recommend aligning training to employment opportunities in each country [18]. In addition, employing healthcare specialists who have a rural background in rural areas and hold training programs rather than sending patients to urban areas would be cost-effective.

### Saudi healthcare workforce in the present and future

SA is set to undergo a huge transformation in the next 10 years due to the iconic Saudi Vision 2030, which will result in the establishment of the new city NEOM and many other major investments. One of the projects is government public–private partnerships. The private sector comprises 25% of the total hospital beds in SA [9]. The government is going to privatize 290 hospitals and 2300 primary care clinics by 2030 and provide comprehensive medical insurance to Saudi citizens [38, 39]. This is going to expand citizens’ choice of healthcare providers. In addition, the government has encouraged foreign investments, which can allow foreign investors full ownership in SA. Privatization, government insurance for citizens, and the fast-growing and aging population of SA will definitely result in an increased demand for various healthcare needs in the future [40]. Alomi et al. concluded that the pharmacology workforce will require 12 078 clinical pharmacists by 2030 [41]. In addition, only 5% of all physicians are family physicians, according to the MOH report. One of the targets of Saudi Vision 2030 is to increase the number of physicians to 1 per 500 people by 2030 [42]. It also aims to increase healthcare services in residential areas from 78 to 88% of all residential areas in SA [42]. Thus, to relieve the work burden on the current workforce; to make up for the current undersupply of healthcare providers, including dermatologists; and to ensure that the country will not face an undersupply in the future, we recommend increasing the capacity of medical education and training programs.

## Limitations

This study sheds light on the dermatology workforce in SA over the last decade. However, this study has some limitations. Data on the ages, work hours, and workloads of the dermatologists would have helped determine if there was a surplus or shortage of dermatologists. Furthermore, there is no national registry of dermatology-related diseases in SA, and as such, there is a lack of knowledge about the incidence and prevalence of such diseases in the country, making it difficult to predict what constitutes a sufficient supply of dermatologists.

Although many studies have provided estimates regarding the requirements for future workforces, we believe that building a similar model is not applicable to our study for many reasons [43, 44]. First, the number of dermatology program graduates in the last 10 years has been inconsistent. In addition, we have no data regarding dermatologists leaving the workforce due to retirement or death. The government is planning to privatize the healthcare system and provide health insurance to all citizens. Thus, citizens will have more choices regarding healthcare providers. We believe that this will shift the burden of providing healthcare to the private sector. The private sector will most likely compensate for any shortages in personnel by employing more healthcare providers or by increasing the working hours of the existing workforce.

## Conclusions

This study revealed that only a quarter of dermatologists in SA are Saudi. In addition, the number of non-Saudi dermatologists has increased in the last 10 years, while the number of Saudi dermatologists in proportion to the population has remained almost constant. There is also a geographic maldistribution of dermatologists in the country, with urban areas having a higher number of dermatologists than rural areas.

We encourage local studies to elucidate dermatologist appointment waiting times, dermatologists’ working hours, and the factors influencing the geographic maldistribution of dermatologists in the country. In addition, we recommend that the SCFHS include a survey before applying a classification system and issuing and renewing medical licenses that account for the required information necessary to estimate the supply needed to meet the demands of the Saudi healthcare system and to build an accurate projection model of future physician numbers. There are seven major programs that are part of Saudi Vision 2030, one of which is the Quality of Life Program [45]. One of the aims of such a program is to improve and develop the workforce in the healthcare sector. To attain this goal, we need to address the previously mentioned challenges, namely, the shortage of Saudi dermatologists, the low capacity of related training programs, and the geographic maldistribution of the dermatology workforce.

## Data Availability

The data sets used and analysed during the current study are available from the corresponding author on reasonable request.

## Abbreviations

SCFHS: Saudi Commission for Health Sciences.
MOH: Ministry of health
SA: Saudi Arabia
IRB: Institutional review board
APA: Advanced physician assistant
